# Nurse’s A-Phase Material Enhance Adhesion, Growth and Differentiation of Human Bone Marrow-Derived Stromal Mesenchymal Stem Cells

**DOI:** 10.3390/ma10040347

**Published:** 2017-03-27

**Authors:** Ruben Rabadan-Ros, Salvador Aznar-Cervantes, Patricia Mazón, Patricia Ros-Tarraga, Piedad N. De Aza, Luis Meseguer-Olmo

**Affiliations:** 1 Grupo de Regeneración y Reparación de Tejidos: Ortobiología, Biomateriales e Ingeniería de Tejidos, Universidad Católica San Antonio de Murcia (UCAM), Guadalupe, 30107 Murcia, Spain; p.ros.tarraga@gmail.com (P.R.-T.); lmeseguer.doc@gmail.com (L.M.-O.); 2 Department of Biotechnology, Instituto Murciano de Investigación y Desarrollo Agrario y Alimentario (IMIDA), La Alberca, 30150 Murcia, Spain; sdac1@um.es; 3 Departamento de Materiales, Óptica y Tecnología Electrónica, Universidad Miguel Hernández, Avda. Universidad s/n, Elche, 03202 Alicante, Spain; pmazon@umh.es; 4 Instituto de Bioingeniería, Universidad Miguel Hernández, Avda. Universidad s/n, Elche, 03202 Alicante, Spain; piedad@umh.es; 5 Servicio de Cirugía Ortopédica y Traumatología del Hospital Clínico Universitario Virgen de la Arrixaca, 30120 Murcia, Spain

**Keywords:** TCP-C_2_S Nurse’s A-phase ceramic, biomaterials, adult human mesenchymal stem cells, solid state reaction, biomedical applications

## Abstract

The purpose of this study was to evaluate the bioactivity and cell response of a well-characterized Nurse’s A-phase (7CaO·P_2_O_5_·2SiO_2_) ceramic and its effect compared to a control (tissue culture polystyrene-TCPS) on the adhesion, viability, proliferation, and osteogenic differentiation of *ah*MSCs in vitro. Cell proliferation (Alamar Blue Assay), Alizarin Red-S (AR-s) staining, alkaline phosphatase (ALP) activity, osteocalcin (OCN), and collagen I (Col I) were evaluated. Also, field emission scanning electron microscopy (FESEM) images were acquired in order to visualise the cells and the topography of the material. The proliferation of cells growing in a direct contact with the material was slower at early stages of the study because of the new environmental conditions. However, the entire surface was colonized after 28 days of culture in growth medium (GM). Osteoblastic differentiation markers were significantly enhanced in cells growing on Nurse’s A phase ceramic and cultured with osteogenic medium (OM), probably due to the role of silica to stimulate the differentiation of *ah*MSCs. Moreover, calcium nodules were formed under the influence of ceramic material. Therefore, it is predicted that Nurse’s A-phase ceramic would present high biocompatibility and osteoinductive properties and would be a good candidate to be used as a biomaterial for bone tissue engineering.

## 1. Introduction

The replacement of lost bone tissue using biomaterials has been an accepted procedure in several surgical disciplines (orthopaedic, oral-maxillofacial, and neural surgery, among others) [1], as a consequence of the drawbacks and effectiveness observed with the use of the autologous bone grafts (widely accepted as the most efficient) and allogenic bone grafts [2]. This fact has led to the development of alternative natural and synthetic materials to obviate the need for bone grafts in the last decades. Tissue engineering can be conceptualised as the use of materials to promote new tissue formation and it involve interactions of the cells with the material [3]. Based on this, bone tissue repair may be one of the major applications of this emergent discipline. The development of bioceramics has provided promising alternatives to replace or increase parts of the skeletal system [4]. Ceramic-based bone graft substitutes include calcium phosphate (CaP) based ceramics such as hydroxyapatite, β-tricalcium phosphate, and bioactive glasses. These materials can be used alone or in combination with other ceramics or compounds as proteins or polymers as polylactic (PLA) or poly lactic-*co*-glycolic acids (PLLA) [5,6]. They have been developed for hard tissue repair due to their biocompatibility and osteoconductivity [7].

Currently, there is a constant need for bone implant formulations with osteointegrative properties. One of the approaches to improve bone stimulating features of these biomaterials is the incorporation of bone stimulator ions into their chemical compositions. Many researches have focused on preparation and characterization of bioceramic-based materials incorporating ions as Si, Ca, Mg, or Zn. Si or Ca in calcium silicate-based materials may play an important role in cell growth on the materials [8,9]. Si is recognised because of its unique effect on osteoblastic differentiation and thus bone mineralization [10].

Bone marrow is a mixture of hematopoietic, vascular, stromal, and mesenchymal cells capable of repairing parts of the skeletal system due to the ability of mesenchymal stem cells (MSCs) to differentiate into mesodermal lineage such as osteoblasts, chondrocytes, adipocytes, miocytes, etc. [11]. Stem cells have the potential to augment the performance of current bone graft substitutes and are the focus of a great deal of ongoing research. Therefore, a bone marrow cell culture system seems to be useful in order to study interactions occurring at the cell-material interface of implants directed to bone tissue engineering.

Compositions belonging to the sub-system Nurse’s A-phase-silicocarnotite within the system Ca_3_(PO_4_)_2_–Ca_2_SiO_4_ (TCP-C_2_S) are promising candidates for preparing ceramic bone implants [12,13,14]. Nurse’s A-phase is a solid solution with an approximate composition of 7CaO·P_2_O_5_·2SiO_2_, which has been recently synthesized at temperatures around 1550 °C in our laboratory and represents a good candidate for biomedical applications [14,15,16,17]. The major goal of the present study was to evaluate the initial response of Si-Ca-P monophasic ceramic called Nurse´s A-phase in its ability to provide a cytocompatible optimal microenvironment for *ah*MSCs to enhance the attachment, adhesion, proliferation, and osteogenic differentiation with a view to using the material for bone tissue engineering in the context of advanced therapy medicinal products (ATMP).

## 2. Results

### 2.1. Inductively Coupled Plasma (ICP)

**Figure 1:**
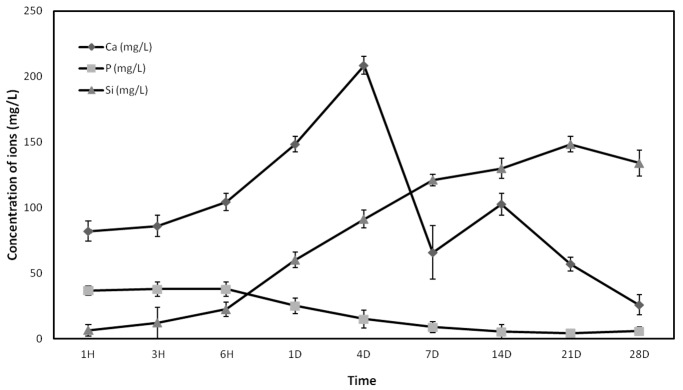
Changes of Si, Ca, and P concentrations of the growth medium (GM) after inmersion of Nurse’s A-phase ceramic for various times.

The release and ionic concentrations of soluble Si, Ca, and P elements by dissolution of the A phase ceramics in relation to time of immersion and analysed by ICP-OES are shown in Figure 1. The results showed a significant initial increase of the calcium (Ca) concentration during the first 24 h of exposure, reaching the maximum concentration (208 mg/L) after four days of immersion. The Ca concentration started to decrease from the fourth day until the last day of the test, when the concentration is minimal. The Phosphorus concentration (P) decreased at day 1 and it was maintained in values of 5 mg/L from the first week to the final of the experiment. The Silicon (Si) concentration increased from the beginning of the experiment, reaching the maximum value of release after 21 days of immersion (148 mg/L).

### 2.2. Field Emission Scanning Electron Microscopy (FESEM)

In order to evaluate adhesion and morphology of *ah*MSCs growing on the Nurse’s A-phase ceramic, the scaffolds cultured with cells were examined by FESEM at days 1, 3, 7, 14, 21, and 28 after the seeding in growth medium (GM) and just at 28 days in osteogenic differentiation medium (OM).

**Figure 2:**
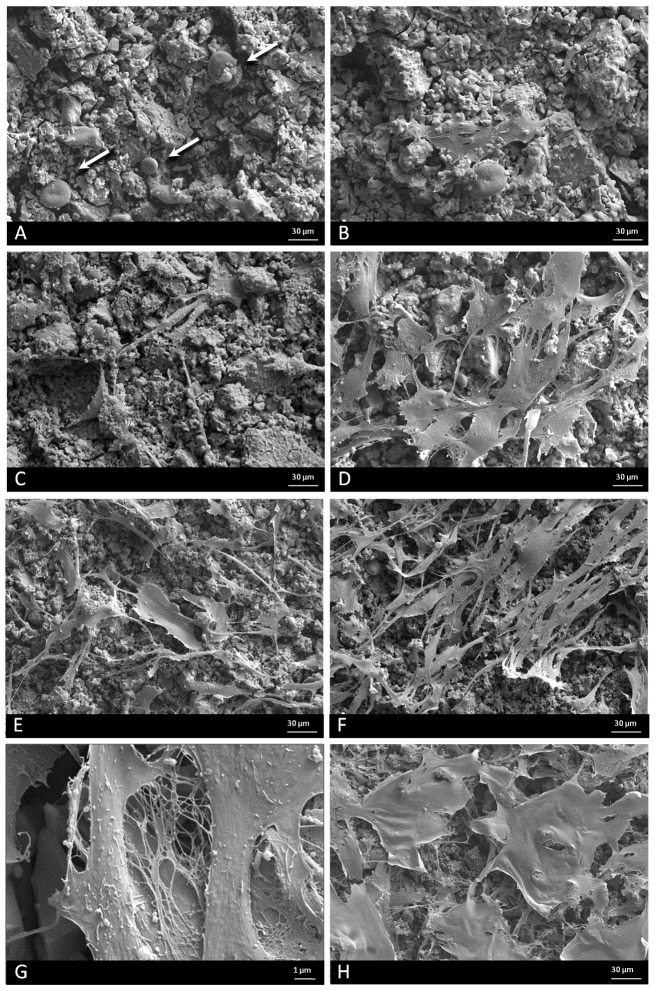
FESEM images of the *ah*MSCs cells grown on ceramic surfaces at 24 h (**A**) (300×) with rounded appearance (arrows), 3 days (**B**) (300×); 7 days (**C**) (300×); 14 days (**D**) (300×); 21 days (**E**) (300×); 28 days (**F**) (300×) in GM and at 28 days in osteogenic differentiation medium (OM) (**H**) (300×) and collagen bundles occupying intercellular gaps at 28 days (**G**) (3000×).

After 24 h in GM, *ah*MSCs seeded on the material surface were observed as individual cells (Figure 2A). Majority of them exhibited spherical or round shape (indicated by arrows), and some of them had a small central depression. The ceramic showed a granular layer composed of spicule-like structures and small nodules.

Three days after the seeding (Figure 2B), the cells showed an initial spreading, but some spherical forms still persisted as in the first day.

By day 7 (Figure 2C), all cells were stretched and they showed some thin cytoplasmatic extensions (filopodia). At this time, the presence of mineralization was also considerable. The nodules suggested the deposition of Nurse’s A-phase material surface. Granules were also observed above cells, in contact with their membranes. This fact can be explained as a consequence of the formation of the nodules during the proliferation of the cells, covering the material and the cellular surfaces. 

After 14 and 21 days (Figure 2D,E, respectively), *ah*MSCs seeded on the material showed a fibroblastic appearance, stretched and covering much of the surface. There were not cytotoxicity signals or morphological alterations throughout the study.

Major cell growth was observed at 28 days. The cells almost coated the whole material surface forming a monolayer and showing a greater number of interconnections. Abundant extracellular matrix expressed as fibrillar network (Figure 2G) was also observed at this time, occupying the intercellular gaps. Morphological differences were observed between cells cultivated with GM (Figure 2F) and OM (Figure 2H). The appearance of the cells treated with GM showed a fibroblastic appearance, while *ah*MSCs that grew with OM had polygonal shapes like osteoblastic cells.

### 2.3. Cellular Metabolic Activity

**Figure 3:**
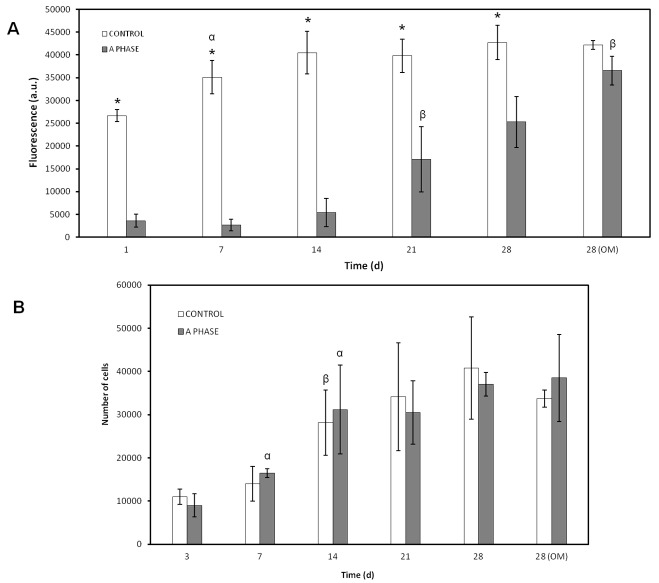
(**A**) Cellular metabolic activity of the cells seeded on the Nurse’s A-phase scaffolds compared to cell cultures on plastic (positive controls) obtained by means of Alamar Blue Assay; (**B**) Proliferation assay of the cells seeded in indirect contact with Nurse’s A-phase compared to cells growing on plastic used as control. (*) denotes significant differences (*p* < 0.05) between Nurse’s A-phase and control at the same experimental time; (α) denotes significant differences (*p* < 0.05) between different experimental times obtained for control samples; (β) denotes significant differences (*p* < 0.05) between different experimental times obtained for cells growing on Nurse’s A-phase scaffolds.

Results of cellular metabolic activity by Alamar Blue are graphically displayed in Figure 3A. These results are shown as fluorescence arbitrary units (a.u.). *ah*MSCs cultured on Nurse’s A-phase ceramic showed lower metabolic activity than control at early stages. The cells seeded on plastic (control) maintained the maximum value of metabolic activity seven days after the seeding.

A significant increase in the cellular metabolic activity was observed for cells cultured on Nurse’s A-phase ceramic. By day 7, the cell metabolic activity raised but there were not significant differences (*t*-test, *p* > 0.05), 2 weeks after the seeding the cellular metabolic activity duplicated his average value (2070.80 a.u.) compared to the results obtained at seven days (882.70 a.u.). At 21 days, the metabolic activity continued growing up and it was significantly higher (*t*-test, *p* < 0.05), and by 28 days, it was registered the highest metabolic activity value for cells growing in OM (*t*-test, *p* < 0.05). Remarkably, *ah*MSCs cultured on Nurse’s A-phase ceramic with OM exhibited major metabolic activity than cells cultured with GM, and there were not significant differences with control at the same experimental time (*t*-test, *p* > 0.05).

### 2.4. Proliferation Assay

As can be observed in Figure 3B, *ah*MSCs seeded in indirect contact with Nurse’s A-phase and cells growing on plastic used as control were counted at different experimental times, using trypan blue staining (in order to confirm their viability), and no significant differences were found between both treatments (*t*-test, *p* > 0.05). Cells proliferated throughout the wells of the culture plates quickly, occupying the entire surface at 14 days. Once this happened, the number of cells remained constant until the end of the experiment (28 days).

### 2.5. Differentiation Assays

#### 2.5.1. Alkaline Phosphatase (ALP) Activity

**Figure 4:**
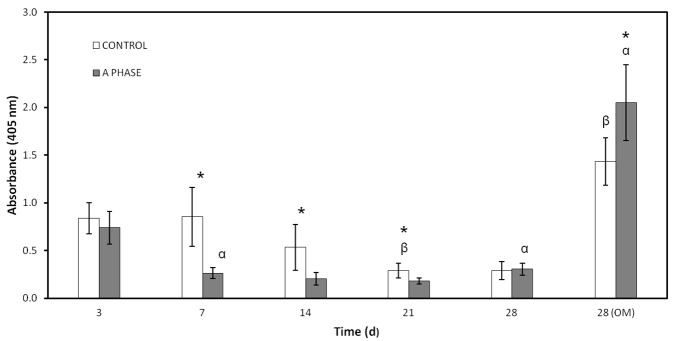
Alkaline phosphatase activity of the cells seeded in indirect contact with A phase compared to plastic used as control. (*) denotes significant differences (*p* < 0.05) between A phase and control at the same experimental time; (α) denotes significant differences (*p* < 0.05) between different experimental times obtained for control samples; (β) denotes significant differences (*p* < 0.05) between different experimental times obtained for cells growing on Nurse’s A-phase scaffolds.

Results of ALP activity measurements obtained from *ah*MSCs cultured in indirect contact of Nurse’s A-phase ceramics compared to plastic are graphically shown in Figure 4 as a function of optical density units (OD). The ALP activity is apparently prevented by Nurse’s A-phase at early stages. In fact, from the day 7 to the day 21, its expression is always significantly lower in the cells cultured on biomaterials compared to the control (*t*-test, *p* < 0.05). The highest ALP activity is observed at 28 days coinciding with the addition of OM due to the induction of the differentiation of *ah*MSCs into osteoblasts. Contrary to the rest of the study, the cells cultured with Nurse’s A-phase are significantly more active than control cells in this case (*t*-test, *p* < 0.05).

#### 2.5.2. Alizarin Red-s (AR-s) Assay

**Figure 5:**
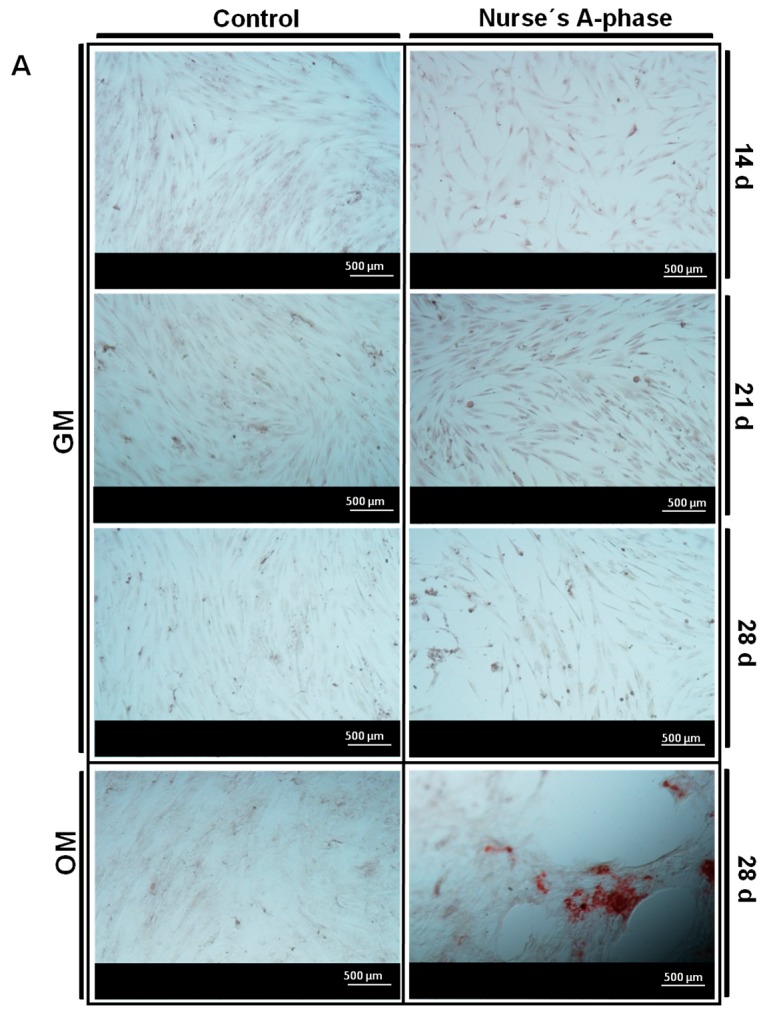

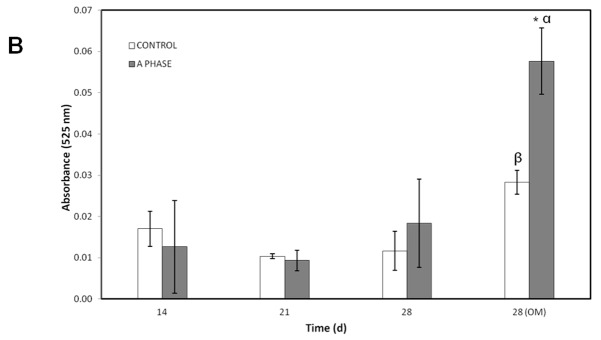
(**A**) AR-s images (×5 magnification) of the *ah*MSCs seeded as control in plastic and cells grown in indirect contact with Nurse’s-A phase ceramic after 14 days, 21 days, 28 days with GM and 28 days in OM; (**B**) AR-s quantification of the cells seeded in indirect contact with A phase compared to control cells growing on plastic without the presence of the material. (*) denotes significant differences (*p* < 0.05) between A phase and control at the same experimental time; (α) denotes significant differences (*p* < 0.05) between different experimental times obtained for control samples; (β) denotes significant differences (*p* < 0.05) between different experimental times obtained for cells growing in indirect contact with Nurse’s A-phase scaffolds.

The potential ability of cells to form calcium-rich mineralized nodules due to the presence of Nurse’s A-phase ceramic discs in indirect contact with the *ah*MSCs was examined by Alizarin Red calcium staining under optical microscopy (Figure 5A) and quantified by spectrophotometry (Figure 5B).

As it is shown in Figure 5A, the control monolayers of *ah*MSCs cultured with GM and OM showed no staining. However, AR-s, *ah*MSCs growing in indirect contact with Nurse’s A-phase scaffolds showed some areas clearly stained, especially intense 21 days after the seeding and until the end of the experiment. When cells were grown with OM at 28 days, the presence of the mineralized nodules dramatically increased. The quantitative examination of data obtained from this analysis confirmed the results of the visual study, with a higher value of OD in cells growing in the presence of A phase discs at 28 days, cultured with OM (*t*-test, *p* < 0.05). 

#### 2.5.3. Osteocalcin Assay

**Figure 6:**
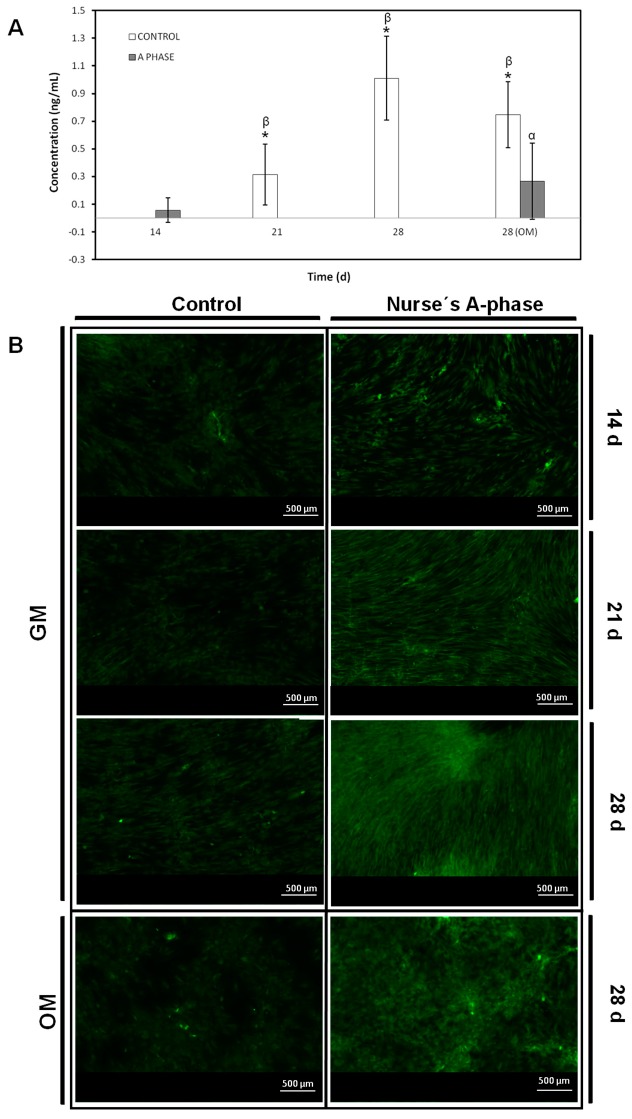
(**A**) Concentration of osteocalcin produced by the cells seeded in indirect contact with A-phase compared to control cells growing on plastic without the presence of the material. (*) denotes significant differences (*p* < 0.05) between A phase and control at the same experimental time; (α) denotes significant differences (*p* < 0.05) between different experimental times obtained for control samples; (β) denotes significant differences (*p* < 0.05) between different experimental times obtained for cells growing in indirect contact with Nurse’s A-phase scaffolds; (**B**) Immunofluorescence staining of Collagen I images (×5 magnification) of the *ah*MSCs seeded as control in plastic and cells grown in indirect contact with Nurse’s A-phase ceramic at 14 days, 21 days, and 28 days under different experimental conditions.

The concentration of osteocalcin (OCN) (ng/mL) released by *ah*MSCs to the culture medium was measured and is represented in Figure 6A. The cells seeded in indirect contact with Nurse’s A-phase only released the OCN to the medium after 28 days of culture by means of induction with OM. However, control cells produced OCN from day 21, until the end of the experiment. There were no significant differences between growing them with OM or GM 28 days after the seeding (*t*-test, *p* > 0.05).

#### 2.5.4. Collagen Type I Expression

Figure 6B shows immunofluorescence staining of Col I from extracellular matrix produced by *ah*MSC. The cells cultured as a control without any other stimulus than the presence of culture plastic are represented in the left column. Apparently, there were no differences between the images, and they seem to emit the same fluorescence from day 14 to day 28 (Figure 6A), even when adding OM. On the other hand, the cells seeded in indirect contact with Nurse’s A-phase scaffolds (right column) showed increasing fluorescence intensity from day 14 until the end of the experiments, with the highest intensity at 28 days, regardless of the type of medium used. However, the arrangement of collagen seems to vary by acquiring rounded shapes using OM.

## 3. Discussion

Future biosynthetic bone substitutes based on natural and synthetic biomaterials may obviate the need for autologous bone grafts, considered today as the gold standard for bone repair [18]. Advances in tissue engineering will generate new carrier constructs that repair, regenerate, and restore tissue to its functional or physiologic state. An ideal biomaterial scaffold designed for bone tissue engineering requires osteoconductive, osteoinductive, and osteogenic properties. In addition, it must be degraded over time into non-toxic products, which can be metabolised by physiological mechanisms.

Adding inorganic elements to scaffolds could significantly improve the bioactivity of materials as previously seen with bioglass, bioceramics that contain CaO, SiO_2_, and P_2_O_5_ or glass-ceramic [19,20]. These constructs are likely to include additional growth factors, evolving biological scaffold and incorporation of undifferentiated MSCs [21].

*ah*MSCs are commonly used to determine the responses of bone cells to biomaterials due to the fact that they are more sensitive to the action of ions delivery than the mouse or rats cells. Thus, for preclinical studies, *ah*MSCs are recommended instead of MSCs from animal origin [22]. The interaction and biological response of bone marrow–derived *ah*MSCs (proliferation and osteogenic differentiation) growing in contact with Nurse’A phase ceramics were investigated in this work.

Nurse’s A material was conditioned by incubation in FBS for 1 h and 24 h prior to cell seeding because it has been indicated that the absorption of serum proteins is effective for cell proliferation [23] and cell adhesion.

In vitro studies are widely employed as preliminary tests to evaluate the biocompatibility of new materials with potential applications in biomedicine [24]. In order to evaluate the biocompatibility of Nurse’s A-phase materials, we explored the attachment and growth of *ah*MSCs on scaffolds by means of FESEM. The cell proliferation was evaluated indirectly by the metabolic activity test Alamar blue and by indirect contact to evaluate the effect of ions released.

The ceramic discs cultured with *ah*MSCs examined by FESEM showed rounded globular cells on the ceramic surfaces at 24 h because they are not adhered yet. The cells were not fully adhered until day 7, when they showed the characteristic flattened polygonal shape and they were metabolically active, displaying higher values of proliferation. It has been demonstrated that levels of Ca concentrations up to 300 mg/L are cytotoxic [25], but lower concentrations are suitable for cell proliferation, differentiation, and extracellular matrix mineralization. The new biomaterial obtained, Nurse’s A-phase, has a high proportion of CaO in its composition, causing the high level of Ca^2+^ ion release in the first week. Low levels of proliferation and rounded cell shape at first stages could be explained due to the high levels of calcium until day 4, when the peak of release was shown. However, the cells seeded in indirect contact with the material did not show a lower metabolic activity than the control cells, so the adaptation to the irregular and shifting surface due to ion exchange should be the most likely cause of the lower cellular activity shown by the Alamar blue assay.

After this adaptation period, the cells showed normal fibroblastic morphology, finally occupying the entire surface of the material and showing levels of metabolic activity similar to the control cells, indicating that Nurse’s A-phase material is not cytotoxic and is highly biocompatible, favouring cell proliferation. From day 21 in culture, an abundant extracellular matrix with aspect of network was synthetized on the surface of all samples in the presence of the A phase materials. In fact, the intercellular gaps were occupied by that network fibrillar material. FESEM images of *ah*MSCs at 28 days cultured with OM displayed polygonal shapes typical for *ah*MSCs undergoing osteogenic differentiation, whereas cells cultured with GM at the same time showed fibroblastic pattern.

The combination of *ah*MSCs and biomaterials is a very important issue for tissue engineering and biomedicine. Thus, an evaluation of the osteogenic differentiation of *ah*MSCs on ceramics by ALP activity assay and calcium deposits staining is crucial. In adult MSCs, a high level of ALP is associated with the process of differentiation rather than with stemness. It is well known that ALP is considered an osteogenic marker expressed at the beginning of *ah*MSCs differentiation but may gradually decrease as the cell differentiate, while calcium nodules formation occurs at late stages [26].

Nurse’s A material clearly seems to inhibit ALP activity, maybe because of the ions released to the environment. The role of ALP in osteoblastic differentiation is to participate in the formation of calcium-phosphate crystals by the release inorganic phosphates to the solution [27]. These crystals begin to form independently from ALP activity in the case of the bioceramics, due to the self-bioactivity of the ceramics, and this may be the cause of the reduction in *ah*MSCs ALP activity. On the other hand, ALP activity increased in cells incubated with A phase materials at 28 days after adding OM, concordant with the maximum release of silicon to the medium. The release of ions may play an important role in these processes and recent studies indicate that silicon promotes cell proliferation [10], ALP expression, and mineralization without affecting the rate of bone formation or bone loss [28]. Also, it has been demonstrated that low concentrations of Ca can stimulate cell differentiation indicated through increased ALP activity, whereas higher contents of Ca tend to decrease ALP stimulation levels. Moreover, osteogenic supplements, like ascorbic acid and β-glycerophosphate, up-regulate ALP gene expression [29]. Osteogenic supplements in OM, low concentrations of Ca, and increasing levels of Si from day 21 to day 28 may create a synergistic effect on the stimulation of ALP activity.

Other evidence of osteogenic differentiation includes mineral formation of *ah*MSCs. The mineralization over time was evaluated with AR-s staining. Control cultures remained colourless and there were no stained areas at any experimental time, while the cells seeded in indirect contact with A phase ceramics showed highly stained areas during the whole experiment and more intense staining at 28 days (especially after adding the OM). Mineralization begins with hydroxyapatite formation in the matrix vesicles budding from osteoblasts. Hydroxyapatite is formed from Ca^2+^ incorporated by the annexin calcium channel and from inorganic phosphate (Pi) [27]. In this case, the release of Ca^2+^ from A phase ceramic is high. The biomaterial provides also phosphorus ions constantly to the medium since the first day and ALP is very active at day 28, contributing this way with more P ions to the hydroxyapatite formation. All these processes explain the early formation of mineralization nodules in the presence of A phase ceramics and represent a potential improvement in the field of biomaterials for bone tissue engineering.

Nearly 90% of the organic matrix of bone is collagen (mainly Col I. Osteoblasts) synthesize, package, and export the organic constituents of bone matrix including Col I and OCN. The major non-collagenous protein produced is OCN, which makes up 1% of the matrix, so Col I and OCN are often used as markers for the bone formation process. Col I synthesis is implicated in first stages of osteogenic differentiation, while OCN is produced in later phases of differentiation (mature) [30]. Ca^2+^ acts through binding to a calcium sensing receptor, inducing the expression of osteoblastic differentiation markers such as Col I and may be the factor responsible to stimulate the intense inmunofluorescence of Col I detected in *ah*MSCs grown in indirect contact with A phase ceramic since early stages. OCN showed very low values until the last measurement (28 days), the result of which may be explained due the late expression of this marker described in the bibliography. 

## 4. Materials and Methods

### 4.1. Porous Nurse’s A-Phase Ceramic Preparation

Porous Nurse’s A-phase ceramic was obtained by a solid-state reaction to form a stoichiometric mixture of calcium hydrogen phosphate anhydrous (CaHPO_4_; Panreac S.L.U., Barcelona, Spain), calcium carbonate (CaCO_3_ > 99.0 wt %; Fluka-Sigma Aldrich Quimica SL, Madrid, Spain) with an average particle size of 13.8 µm, and silicon oxide (SiO_2_ > 99.7 wt %; Strem Chemicals UK Ltd., Cambridge, UK) with an average particle size <50 µm, The details of the technique and the characterization of the ceramic can be found in previous publications [22,23,24].

Discs-shaped Nurse’s A-phase samples, measuring 7 mm in diameter and 3 mm in length, were cut from the sintered bulk with a diamond saw. Finally, Nurse’s A-phase discs were individually packaged and sterilized by gas-plasma (Sterrad-1005TM, ASP, Irvine, CA, USA).

### 4.2. Inductively Coupled Plasma Optical Emission Spectroscopy (ICP)

The concentration of calcium (Ca), silicon (Si), and phosphorus (P) ions released from the Nurse’s A-phase ceramic was determined by inductively coupled plasma optical emission spectroscopy (ICP-OES, PerkinElmer Optima 2000, Waltham, MA, USA). Five Nurse’s A-phase discs were placed in a 48-well plate and 1 mL of growth medium (GM) which consisted of DMEM (Dubelcco´s Modified Eagle Medium) (Sigma-Aldrich, St. Louis, MO, USA) containing 10% FBS (Fetal Bovine Serum) (Sigma-Aldrich, St. Louis, MO, USA) and 100 g·mL^−1^ penicillin-streptomycin (Sigma-Aldrich, St. Louis, MO, USA) was added to each well, keeping the plates in a humidified atmosphere of 95% air, 7.5% CO_2_ at 37 °C. The solutions were extracted to estimate ions concentration for 1 h, 3 h, 6 h, 1 day, 4 days, 7 days, 14 days, 21 days, and 28 days and immediately replaced by fresh GM.

### 4.3. Isolation, Characterization and Culture of Adult Human Bone Marrow-Derived Mesenchymal Stem Cells (ahMSCs)

The isolation and characterization of undifferentiated multipotent *ah*MSCs was described previously [31]. All the procedures were approved by the Institutional Ethical and Clinical Trials Committee (V. Arrixaca University Hospital of Murcia, Murcia, Spain). Briefly, bone marrow biopsy (40 mL) was aspirated from the iliac crest of three healthy male human volunteers aged 25–40 years. Informed consents were obtained from all of them. *ah*MSCs were isolate from the bone marrow based in their propensity to adhere to the bottom of standard tissue culture flasks (Nunc, Thermo Fisher, Geel, Belgium) and cultured in GM in a humidified atmosphere of 95% air, 7.5% CO_2_ at 37 °C for three weeks. Initially, the medium was changed after 48 h to remove non-adherent cell and subsequently renewed three times a week. After 10 days in primary culture, when *ah*MSCs reached 85%–90% confluent, they were detached with 0.25% solution trypsin-EDTA (Sigma-Aldrich, St. Louis, MO, USA), centrifuged at 300 rpm for 10 min and subcultured in an initial density of 5 × 10^3^ cell·cm^−2^ in 75 cm^2^ flasks. Cells have been characterized following the criteria of International Society of Cell Therapy (ISCT) [32] (data not shown).

After cell expansion, *ah*MSCs of passage 3 (P3) were used for all experiments.

### 4.4. Field Emission Scanning Electron Microscopy

Cell adherence, distribution, morphology, and proliferation of the *ah*MSCs studies were performed to investigate the biofuncionality of the material at 1, 3, 7, 14, 21, and 28 days after the seeding. *ah*MSCs were seeded onto the tops of disc-shapped Nurse’s A-phase at a density of 5 × 10^3^ cells·cm^−2^ placed in 48 well-plate and cultured with GM at 37 °C, 7.5% CO_2_ in a humidified incubator. Some of the *ah*MSCs cell cultures were also induced to differentiate replacing the GM by MSC osteogenic differentiation medium (OM) at 21 days, which consisted of the GM supplemented with l-ascorbic acid-2-phosphate (0.2 mM; Sigma-Aldrich, St. Louis, MO, USA), dexamethasone (10 nM; Sigma), and β-glycerolphospathe (10 nM; Merck, Darmstadt, Germany). GM or OM was replaced every three days. After the incubation period, the cell-cultured disks or construct were rinsed with phosphate buffer solution (PBS) for 10 min and fixed for 1 h with 3% glutaraldehyde in 0.1 M cacodylate buffer, then were post fixed with 1% osmium tetroxide. Then, the cell-cultured constructs were thoroughly rinsed with PBS and dehydrated in a graded series of ethanol solutions (30%, 50%, 70%, 90%, and 100% *v*/*v*). The critical point drying was undertaken with liquid CO_2_. Finally, specimens were sputter-coated with gold and examined by Field Emission Scanning Electron Microscopy (Merlin™ VP Compact, Carl Zeiss Microscopy S.L., Oberkochen, Germany).

### 4.5. Cellular Metabolic Activity

In order to evaluate the biological properties of the Nurse’s A-phase material in terms of influence in the metabolic activity of the cells Alamar Blue assay was used (Alamar Blue; Invitrogen, Carlsbad, CA, USA). Metabolic activity is commonly used as an indirect measure of cell proliferation. This assay was based on the redox activity of living cell and the metabolic activity was assessed by fluorescence measurement. Briefly, cells were seeded on biomaterial disks in 48-well plates at a cell density of 5 × 10^3^ cells·cm^−2^ and incubated in the same culture conditions described above. In addition, *ah*MSCs were seeded onto tissue culture-treated polystyrene (TCPS) culture plate served as a positive control. Alamar Blue assay was performed at 1, 7, 14, 21, and 28 days after the seeding. ahMSCs were also induced to osteoblastic differentiation replacing GM by OM at 21 days. Medium was replaced every three days. At the end of the culture period, the medium was discarded and the wells were washed twice with phosphate buffer solution (PBS). Each well was filled with 200 μL of fresh medium containing 10% (*v*/*v*) of Alamar Blue reagent and were incubated at 37 °C for 4 h. The reacted dissolution was then transferred to a 96-well plate and the fluorescence measurements were determined directly in a Synergy MX ultraviolet visible (UV-Vis) (Bio Tek Instruments Inc., Winooski, VT, USA) at excitation and emission wavelengths of 560 nm and 590 nm, respectively. The assay was performed at least in triplicate. Results are reported as arbitrary units (a.u.).

### 4.6. Proliferation Assay

To measure cell proliferation and density in indirect contact with Nurse’s A-phase, scaffolds were placed in transwell inserts in 24-well plates and *ah*MSCs were seeded at the bottom of each well at a density of 5 × 10^3^ cells·cm^−2^ and incubated in GM at 37 °C in a humidified atmosphere consisting of 95% air and 7.5% CO_2_. *ah*MSCs were also induced to osteoblastic differentiation by replacing GM by OM at 21 days. The trypan blue solution (0.4%) (Thermofisher Scientific, Waltham, MA, USA) is routinely used as cell staining to assess cell viability based on the concept that viable cells do not absorb waterproof dyes. Living cells were counted using a Neubauer chamber at 3, 7, 14, 21, and 28 days. Others *ah*MSCs were seeded onto plastic in order to have a positive control.

### 4.7. Differetiation Assays

The ability of ahMSCs to differentiate into osteoblasts was studied by alkaline phosphatase (ALP) activity, osteocalcin (OCN) and collagen type I (Col I) production, and formation of nodules of mineralization (in vitro). ALP and Col I are both initial osteogenic markers such that their value may increase in the beginning of osteogenic differentiation. OCN and mineralization are late osteogenic markers.

To carry out all these indirect studies, Nurse’s A phase scaffolds were placed in transwell inserts in 24-well plates and *ah*MSCs were seeded at the bottom of the wells at a density of 5 × 10^3^ cells·cm^−2^ and incubated at 37 °C in a humidified atmosphere consisting of 95% air and 7.5% CO_2_.

#### 4.7.1. Alkalne Phosphatase (ALP) Activity

ALP is an enzyme responsible for removing phosphate groups from many types of molecules. It is expressed by cells during osteogenesis and it is well established as an osteogenic differentiation marker [26]. For these studies the measurements were taken in triplicate at 3, 7, 14, 21, and 28 days after the seeding. Also, *ah*MSCs were induced to osteoblastic differentiation replacing GM by OM at 21 days. *ah*MSCs were also seeded on plastic culture plates in order to have a control of differentiation to osteoblasts without any other stimulus than the presence of OM.

ALP was measured by Quantitative Alkaline Phosphatase ES Characterization Kit (Millipore, Billerica, MA, USA). To quantify ALP activity, the cells were treated following the manufacturer’s protocol and the absorbance of enzyme product was measured at 405 nm on a spectrophotometer (Bio Tek Instruments Inc., Winooski, VT, USA).

#### 4.7.2. Alizarin Red-s (AR-s) Assay

The presence of calcium deposition or nodules of mineralization was evaluated by the specific binding of Alizarin Red (Osteogenesis Quantitation Kit, Millipore, Billerica, MA, USA) to calcium deposits. For this purpose the measurements were taken in triplicate at 14, 21 and 28 days after the seeding. *ah*MSCs were also induced to differentiate replacing GM by MSC osteogenic differentiation medium (OM) at 21 days. After culturing the cells, they were fixed 10 min with 8% paraformaldehyde and stained for 30 min at RT with Alizarin Red Stain Solution. Stained areas were visualized using a Nikon Eclipse 50i microscope (Nikon, Melville, NY, USA), and some photographs were taken using a Nikon *DS*-*Fi1* digital microscope camera (Nikon, Melville, NY, USA). To quantify matrix mineralization, the samples were treated following the manufacturer’s protocol and the absorbance of Alizarin Red S extracts was measured at 525 nm on a spectrophotometer (Bio Tek Instruments Inc., Winooski, VT, USA).

#### 4.7.3. Osteocalcin (OCN) Assay

The OCN production was determined in supernatants using Osteocalcin ELISA kit (Thermofisher Scientific, Waltham, MA, USA) following the manufacturer’s instructions. For this purpose, serum was removed from the culture medium five days before carrying out the determinations. The medium was collected at days 7, 14, 21, and 28 and kept at −20 °C until analysis following manufacturer’s protocol. Three determinations of each simple and standard kit were carried out.

Briefly, 25 µL of medium was pipetted out into microtiter plate coated with anti-osteocalcin antibody followed by 100 µL of anti-osteocalcin labelled with HRP (horseradish peroxidase) and incubated for 2 h at room temperature. After washing with wash solution, 100 µL of chromogenic solution was added to each well for 30 min at room temperature followed by the addition of 100 µL of stopping solution into each well. The absorbance was read at 405 nm (Bio Tek Instruments Inc., Winooski, VT, USA). A calibration curve was plotted and the OCN concentration in sample was determined by interpolation from the calibration curve.

#### 4.7.4. Collagen Type I Expression

Col I expression was tested by immunofluorescence staining at 14, 21, and 28 days. *ah*MSCs were stained after being induced to differentiate by replacing GM by OM at 21 days (28 OM). Briefly, cells were washed three times with PBS, fixed, and permeabilized with cooled Methanol–Acetone solution (1:1) at 20 °C for 10 min. Samples were then blocked with a blocking solution (0.1% TRITON, 0.3% BSA, 10% FBS in PBS 1×) for 30 min at 37 °C. Labelling was performed by incubating the primary antibody in a blocking solution at 4 °C over night. Next samples were incubated with secondary antibody in a blocking solution for 1 h at RT. Then samples were washed with deionized water and mounted. Samples were visualized using a Nikon Eclipse 50i fluorescence microscope (Nikon, Melville, NY, USA) with excitation filters of 494 nm (green, Calcein) and some photographs were taken using a Nikon *DS*-*Fi1* digital microscope camera (Nikon, Melville, NY, USA).

## 5. Calculation

Quantitative data are presented as means ± standard deviation (SD) of at least three specimens per treatment condition. *t*-student test was used for comparison between groups. A *p*-value of less than 0.05 was considered statistically significant.

## 6. Conclusions

This work constitutes the first step to evaluate the potential use of a novel Nurse’s A-phase material as a scaffold for bone tissue engineering. The results stated in terms of biocompatibility, adhesion, and proliferation of *ah*MSCs prove that this A phase ceramic is not cytotoxic and stimulates optimal growth of the cells comparable to *ah*MSCs cultured on plastic.

The expression of the studied osteogenic markers was always more intense in cells growing under the influence of A phase materials itself, but especially when OM was included in the culture conditions. These promising results make Nurse’s A material a promising candidate to be employed as a new bone graft substitute or scaffold for bone tissue engineering.

Future work will be focused on in vivo studies and improvements of the material in terms of composition, by means of the incorporation of different molecules such as growth factors or some biocompatible polymers.
